# Two Structural Designs of Broadband, Low-Loss, and Compact TM Magneto-Optical Isolator Based on GaAs-on-Insulator

**DOI:** 10.3390/nano14050400

**Published:** 2024-02-21

**Authors:** Li Liu, Wan-Ting Chen, Jia Zhao, Chen Zhang

**Affiliations:** School of Information Science and Engineering, Shandong University, Qingdao 266237, China; liulisrzh@163.com (L.L.); 202234052@mail.sdu.edu.cn (W.-T.C.)

**Keywords:** optical isolator, non-reciprocal phase shift, magneto-optical, Mach–Zehnder interferometer, GaAs-on-insulator, multi-mode interferometric coupler

## Abstract

Integrated optical isolators are important building blocks for photonic integrated chips. Despite significant advances in isolators integrated on silicon-on-insulator (SOI) platforms, integrated isolators on GaAs-on-insulator platforms are rarely reported. In this paper, two structural designs of optical isolators based on the TM basic mode of GaAs-on-insulator are proposed. The non-reciprocal phase shift (NRPS) of GaAs/Ce:YIG waveguides with different geometric structures are calculated using numerical simulation. The isolators achieve 35 dB isolation bandwidths greater than 53.5 nm and 70 nm at 1550 nm, with total insertion losses of 2.59 dB and 2.25 dB, respectively. A multi-mode interferometric (MMI) coupler suitable for these two structures is proposed. In addition, suitable manufacturing processes are discussed based on the simulated process tolerances.

## 1. Introduction

Optical isolators in non-reciprocal optical devices are important components for photonic integrated circuits (PICs). They resemble electronic diodes in many ways, except for having an asymmetric scattering matrix that can break the Lorentz reciprocity effect [1]. This unique characteristic enables precise control over unidirectional light transmission, allowing optical isolators to be utilized to prevent reflected light and thereby protect active devices [1,2,3,4,5]. Optical isolators are achieved through spatio-temporal modulation [6,7], nonlinear optical effects [8,9], and magneto-optical (MO) effects [10,11,12,13,14]. MO isolators, benefiting from their entirely passive operation, simple device structure, and large dynamic range, find extensive use in commercial applications for non-reciprocal devices. Non-reciprocal phase shift (NRPS) is a critical technique for realizing MO effects. Applying a magnetic field perpendicular to the direction of light propagation on a MO waveguide induces NRPS [15]. Doping yttrium iron garnet (YIG) with cerium (Ce) or bismuth (Bi), exhibiting a substantial Faraday rotation angle, results in significant NRPS, effectively reducing the device’s footprint [16,17,18,19]. The Ce:YIG thin film, on the other hand, offers several advantages over Bi:YIG, including a six-fold increase in the Faraday rotation coefficient under the same conditions, a smaller temperature coefficient of the Faraday rotation angle, and a higher cost-effectiveness, making it a research hotspot in the field [20,21]. These advancements lay the foundation for achieving low loss, wide isolation bandwidth, and compact MO isolators.

Currently, the reported integrated optical isolators are primarily based on silicon-on-insulator (SOI) platforms. Utilizing wafer bonding or thin-film deposition techniques, high-performance MO isolators and rings on SOI substrate have achieved remarkable results [10,22,23,24,25]. These devices exhibit an isolation ratio of up to 25 dB [24], with an insertion loss reduced to 4 dB [10]. SOI waveguides garnered attention due to several key advantages within the communication window around wavelengths of 1.3 and 1.55 µm. They possess a high-efficiency index, low propagation losses [26], and are compatible with CMOS technology. However, SOI waveguides still face limitations in fully integrated nonlinear photon circuits. Silicon (Si), being an indirect bandgap material, is not an efficient light-emitting material. The central symmetry of its crystal structure leads to strong two-photon absorption and free-carrier absorption at critical communication windows.

The gallium arsenide (GaAs) waveguide is characterized by high index contrast and a substantial nonlinear coefficient. Its high refractive index leads to stringent mode confinement within the waveguide. Recently, GaAs has garnered considerable attention as a preferred platform for on-chip nonlinear optical applications. The distinguishing advantage of GaAs lies in its efficacy as a single-photon source within photonic integrated circuits (PICs) [27,28,29,30]. This presents a compelling opportunity to seamlessly integrate nonlinear optical elements and laser pump sources on a unified platform. Noteworthy GaAs-based photonic devices, including electro-optic switches [31] and single-photon detectors [32], have been extensively documented. Integrated GaAs ring resonators are achieved by bonding GaAs thin-film wafers to insulating substrates [33]. Ce:YIG growth techniques involving altering the garnet composition to facilitate GaAs growth [34] and utilizing sputtered MgO as a buffer layer on a GaAs substrate [35] have been successfully demonstrated. The application of the diluted magnetic semiconductor Cd1-xMnxTe as an isolator on a GaAs substrate yielded isolation exceeding 20 dB at wavelengths between 715 and 735 nm [36]. Furthermore, recent experiments underscored the potential of GaAs on an insulator platform to attain losses comparable to silicon-on-insulator (SOI), with propagation losses of approximately 4 dB/cm [37]. This provides an opportunity to design high-performance GaAs devices on insulator platforms.

However, to date, there remains a dearth of simulation and experimental results for integrated optical isolators based on GaAs-on-insulator. Here, we simulate two fundamental TM mode optical isolators utilizing an asymmetric MZI type on the GaAs-on-insulator platform. Two distinct isolator structures are designed, each comprising two 1 × 2 MMI couplers, NRPS waveguide, and reciprocal phase shift (RPS) waveguide. The MO waveguide is GaAs/Ce:YIG film. The variation of NRPS with different thickness and width of the waveguide was calculated. The maximum NRPS for both structures reaches 9000.39 rad/m, surpassing currently reported values by two to four times [10,13,22,23,24,25]. This results in a compact waveguide size of only 174.53 μm, effectively addressing the size challenges of optical isolators. The isolation is 35 dB, the bandwidth exceeds 70 nm, and the loss is below 3 dB. Low-loss MMI couplers are designed according to different MO waveguide structures. In addition, the effect of the device geometry on the isolation bandwidth and manufacturing tolerances is discussed. Finally, based on the calculated structure tolerance, a reasonable manufacturing process suitable for these two devices is proposed.

## 2. Device Structure and Principle

**Figure 1:**
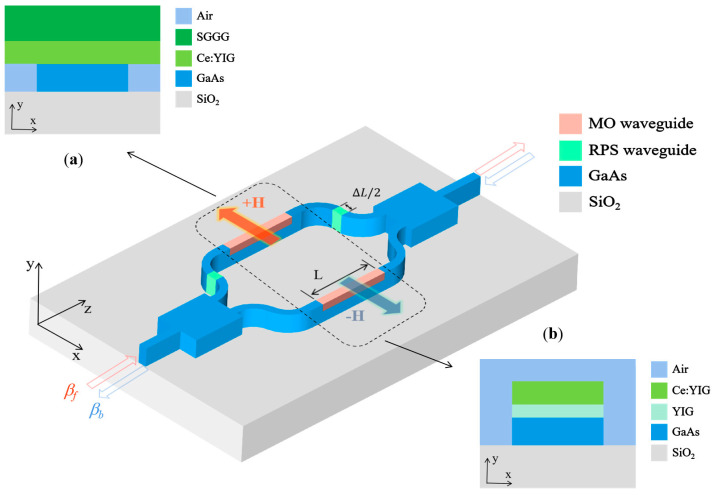
Schematic diagram of an MZI MO isolator proposed on GaAs-on-insulator. MO waveguides have two structures: (**a**) structure A and (**b**) structure B.

Tolerances in the manufacturing process make it difficult to achieve phase matching in TE-TM mode. The MZI-type optical isolator operates in a single-polarization mode to overcome this challenge. Considering two manufacturing processes of wafer bonding and thin-film deposition, we designed structure A and structure B of MZI optical isolators on an insulator platform to achieve optimal device performance. Structure A and structure B are shown in Figure 1. The device consists of two 3 dB 1 × 2 MMI couplers, NRPS waveguides, and RPS waveguides. A cross-sectional image of structure A of the MO waveguide is shown in Figure 1a. It has an asymmetrical refractive index distribution in the y direction and consists of 4 layers, including the GaAs layer (*n*_GaAs_ = 3.3) [38] on the insulator (*n*_SiO_2__ = 1.45) [39] and the Ce: YIG (*n*_Ce:YIG_ = 2.22) [40] layer grown on gadolinium gallium garnet (SGGG), (*n*_SGGG_ = 1.97) [41]. Structure B, depicted in Figure 1b, Ce: YIG containing the seed layer YIG (*n*_YIG_ = 2.22), is deposited directly on the GaAs layer. Notably, a substantial refractive index contrast (*Δn* ≈ 0.40) between the core layer (i.e., GaAs) and the substrate (i.e., SiO_2_) ensures a robust transverse optical mode constraint.

As shown in Figure 1, light waves propagate along the *z*-axis. Light applies a static magnetic field in the *x* direction on the plane of the MO film to saturate its magnetization intensity. In TM mode, the asymmetric permittivity tensor obtained can be expressed as:

(1) $$\varepsilon_{\pm }=\left[\begin{array}{ccc} \varepsilon_{xx} & 0 & 0\\ 0 & \varepsilon_{yy} & \pm j\gamma \\ 0 & \mp j\gamma & \varepsilon_{zz} \end{array}\right]$$

where $\varepsilon_{xx}=\varepsilon_{yy}=\varepsilon_{zz}=n_{Ce:YIG/YIG}^{2}$, and the MO effect is represented by the non-diagonal element *γ*, which is related to the Faraday rotation constant *θ_F_*. The expression for *γ* is:

(2) $$\gamma =\frac{2n_{Ce:YIG/YIG}\theta_{F}}{k_{0}}$$

where *k*_0_ is the wavenumber in a vacuum. For calculations, it is assumed that the *θ_F_* value of Ce: YIG at a wavelength of 1550 nm is −5900 deg/cm [13], while YIG is 200 deg/cm [42]. The corresponding non-diagonal elements *γ* are 1.118 × 10^−2^ and 3.789 × 10^−4^.

The single-mode waveguide excites the basic TM mode in the waveguide region. Due to the influence of the non-diagonal term, the propagation constant in the magnetized waveguide differs from that in the reciprocal waveguide. The forward propagation constant *β_f_* and the reverse propagation constant *β_b_* are distributed in opposite directions. Controlling the direction of light propagation or the applied static magnetic field allows for the manipulation of the magnetization direction of the MO material. The light wave experiences phase changes of *β_f_* and *β_b_*, respectively. This NRPS is defined as the difference between *β_f_* and *β_b_*, which is derived from perturbation theory [15]:

(3) $${NRPS}_{TM}=\beta_{f}-\beta_{b}=\frac{2\beta }{\omega \varepsilon_{0}P}\iint \frac{\gamma }{n_{0}^{4}}H_{x}\partial_{y}H_{x}dxdy$$

where *ω* represents the frequency, and *ε*_0_ is the vacuum permittivity. *P* denotes the magnitude of the power flow in the cross-section. As expressed in (3), the NRPS of TM mode depends on the gradient distribution of *H_x_* components in the magneto-optical material layer along the *y* direction, typically manifested in the asymmetry of the upper and lower cladding materials.

By adjusting the MO effect and the length of the phase shifter, the NRPS provides a phase difference $\left(\beta_{f}-\beta_{b}\right)\times L=\mp \pi /2$ in the ±*x*-axis direction (where *L* is the length of the MO waveguide). The RPS induces a phase shift due to the length difference between the two arms, given by $\beta_{0}\times ∆L=\pi /2+2m\pi$, where *β*_0_ is the propagation constant of light in the GaAs waveguide, Δ*L* is the length difference between the arms, and *m* is an integer, as shown in Figure 1. Phase bias is achieved by adjusting the optical path difference between the two waveguide arms. When the light is directed in the *x* direction, the total phase difference is 2*mπ*. The input light generates phase construction interference in the right coupler and propagates through the device. The total phase difference is (2*m* + 1)*π* when the light is directed in the -*x* direction, leading to destructive interference at the left coupler, and most of it shifts to radiation mode. Therefore, the device design produces constructive interference in front light and destructive interference with reverse light to achieve optical isolation.

## 3. Results and Discussion

### 3.1. Calculation of the NRPS of the MO Waveguide

The NRPS scales with the length of the non-reciprocal waveguide, which affects the compactness of the device. Therefore, optimizing NRPS and the waveguide length is key to designing the device. To obtain a sufficiently large NRPS, we made the lower surface of Ce: YIG at the strongest position of *H_x_* and the upper surface at the edge of the modality. We used the finite element method (FEM) to calculate the mode field distribution and NRPS of two MO waveguide structures. For the simulation, we partitioned the structures into 572,080 domain elements, 73,068 boundary elements, and 1866 edge elements. This density ensures the accuracy of the NRPS values obtained. The single-mode (TM_0_) structure of the waveguide is optimized. Single-mode transmission is especially important for individual optics to avoid negative effects such as mode-to-mode coupling and dispersion.

**Figure 2:**
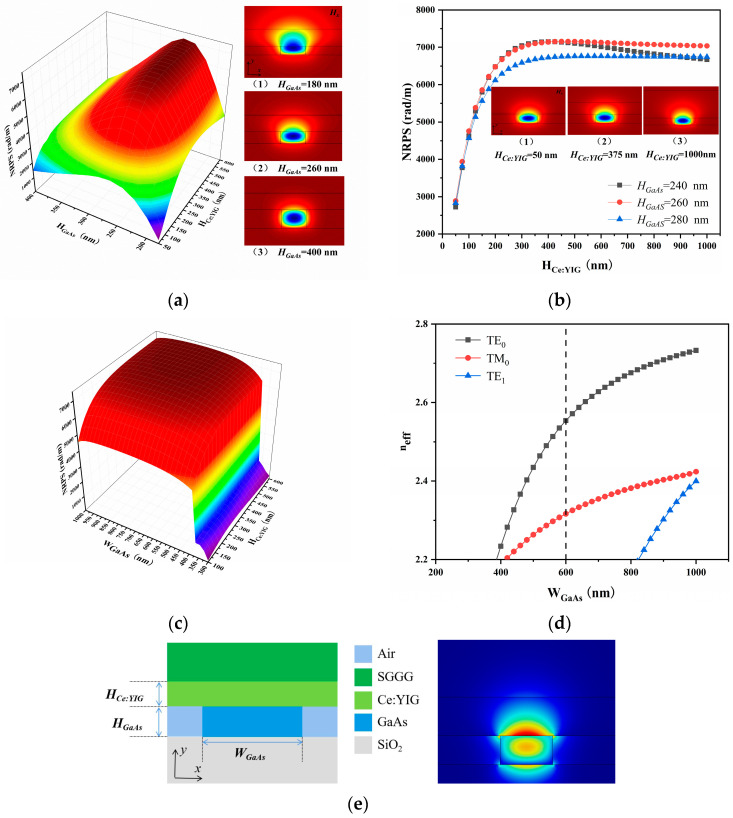
Geometric parameters of structure A. (**a**) When *W*_GaAs_ = 500 nm, NRPS for different height combinations of GaAs/Ce:YIG and *H_x_* field distribution in TM_0_ mode. (**b**) *H*_GaAs_ = 240, 260, or 280 nm, the relationship between *H*_Ce:YIG_ and NRPS. (**c**) When *H*_GaAs_ = 260 nm, NRPS with different *W*_GaAs_ and *H*_Ce:YIG_. (**d**) An effective index profile plot of the waveguide modes as a function of the width. The dashed line represents that when *W*_GaAs_ = 600 nm, the waveguide is in the TM_0_ mode. (**e**) Geometry of structure A and transverse electric field mode field distribution.

Figure 2a illustrates the NRPS variations of the GaAs/Ce:YIG waveguide with different height combinations at 1550 nm, given the waveguide width *W*_GaAs_ = 500 nm and waveguide length *L* = 3 μm in structure A. (In determining the final value of *L*, this length is used throughout). The under-cladding, with a refractive index of 1.45, is sufficiently thick during the simulation to ignore the influence of the substrate. When the MO waveguide thickness *H*_Ce:YIG_ is fixed, NRPS initially increases and then decreases. The corresponding *H_x_* field distribution in TM_0_ mode is depicted in (1), (2), and (3) in Figure 2a. When *H*_GaAs_ = 180 nm, the majority of the *H_x_* field is localized in the upper Ce: YIG layer, with some entering the SGGG layer. At *H*_GaAs_ = 400 nm, almost the entire *H_x_* field is localized in the GaAs waveguide. This results in a small NRPS. For the TM_0_ mode in (2) of Figure 2a, unlike the previous two, the integrated area of the *H_x_* field in Ce:YIG increases when *H*_GaAs_ = 260 nm, obtaining a large NRPS. According to expression (3), the $H_{x}\partial_{y}H_{x}$ term is consistently positive. Considering that Ce: YIG has a negative *γ* value, this results in a negative NRPS value.

To comprehensively explore NRPS variation, *H*_Ce:YIG_ ranging from 50 to 1000 nm was calculated for a core thicknesses of 240, 260, and 280 nm. As shown in Figure 2b, NRPS tends to flatten out after *H*_Ce:YIG_ = 350 nm. Analyzing the magnetic field distribution, it becomes evident that as *H*_Ce:YIG_ increases, the *H_x_* field remains entirely within the GaAs/Ce:YIG layer without significant changes. When *H*_GaAs_ = 260 nm and *H*_Ce:YIG_ = 375 nm, the maximum NRPS is 7128.152 rad/m. Subsequently, NRPS at various *H*_Ce:YIG_ for different *W*_GaAs_ with *H*_GaAs_ = 260 nm was calculated using the same numerical simulation method, as shown in Figure 2c. The NRPS value can reach more than ~5000 rad/m after *W*_GaAs_ = 460 nm. The *H*_Ce:YIG_ has little effect on it. At *W*_GaAs_ = 680 nm and *H*_Ce:YIG_ = 550 nm, the structure achieves a peak NRPS value of 7339.046 rad/m. Considering the accuracy of actual processing, *W*_GaAs_ = 600 nm and *H*_Ce:YIG_ = 350 nm were chosen, resulting in NRPS = 7235.746 rad/m. The corresponding MO waveguide length is 217.09 μm. Despite a reduction of 103.318 rad/m compared to the maximum value, it has a negligible impact on the overall waveguide length when accounting for manufacturing tolerances. An effective index profile plot of the waveguide modes as a function of the waveguide width was studied, as shown in Figure 2d. The waveguide is in a TM_0_ single-mode condition. Figure 2e presents the final structure A diagram and the corresponding transverse electric field mode distribution.

**Figure 3:**
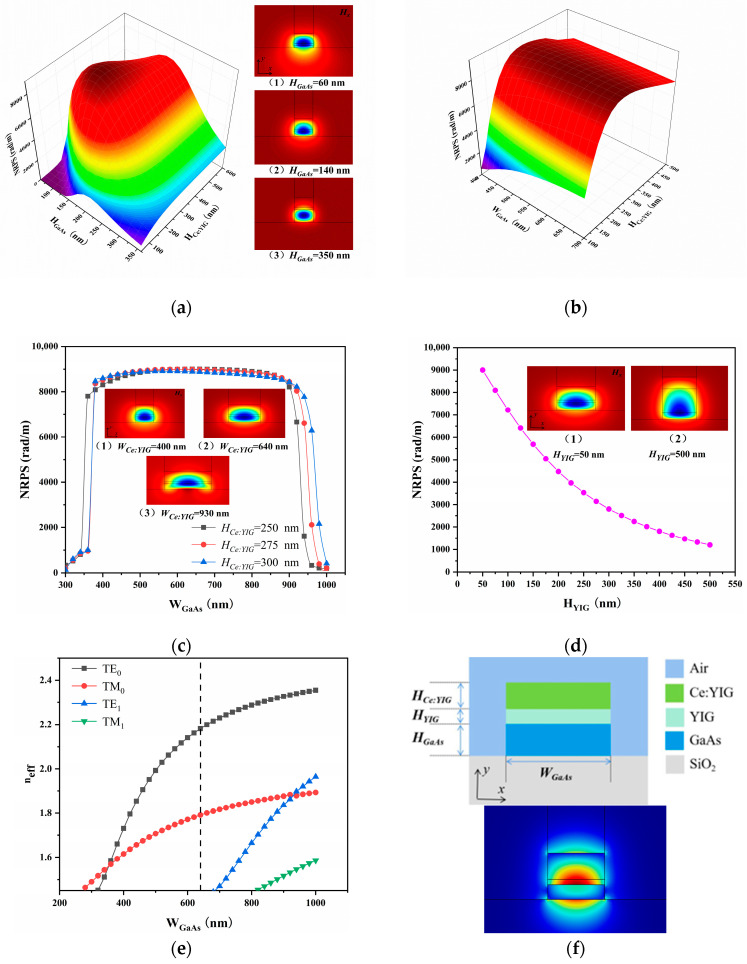
Geometric parameters of structure B. (**a**) When *W*_GaAs_ = 500 nm, NRPS for different height combinations and *H_x_* field distribution. (**b**) When *H*_GaAs_ = 140 nm, NRPS with different *W*_GaAs_ and *H*_Ce:YIG_. (**c**) *H*_Ce:YIG_ = 250, 275, or 300 nm, the relationship between *W*_Ce:YIG_ and NRPS. (**d**) NRPS varies with seed layer *H*_YIG_. *H_x_* is distributed in (1) and (2), respectively. (**e**) An effective index profile plot of the waveguide modes as a function of the width. The dashed line represents that when *W*_GaAs_ = 640 nm, the waveguide is in the TM_0_ mode. (**f**) Geometry of structure B and transverse electric field mode field distribution.

In addition, we calculated the NRPS and *H_x_* distribution of structure B, considering various height combinations and combinations of height and width in TM mode, as shown in Figure 3a,b. In the simulation, the waveguide length *L* is set to 3 μm, same as for structure A. The trend of NRPS with different height combinations aligns with that of structure A. From the *H_x_* distribution in Figure 3a, it becomes apparent that thicker waveguides will limit more electromagnetic fields, resulting in a reduction of the $H_{x}\partial_{y}$ in MO materials. For structural parameters *H*_GaAs_ = 140 nm, *H*_Ce:YIG_ = 275 nm, *W*_GaAs_ = 500 nm, the simulated maximum NRPS is 8922.130 rad/m. Simultaneously, *H*_Ce:YIG_ = 250, 275, or 300 nm were also calculated, and the NRPS with a MO film thickness ranging from 300 nm to 1000 nm is presented in Figure 3c. Likewise, the coupling efficiency of structure B rapidly increases to the maximum, further indicating the insensitivity of NRPS in TM mode to the *x*-direction gradient distribution of *H_x_* components in the MO material layer. With a smaller width, the MO material region shrinks, causing a reduction in the integrated area and subsequently leading to a decrease in NRPS. A larger width results in the main concentration of the mode in the core layer region, reducing the field gradient in the MO layer, which also leads to a decrease in NRPS.

The consideration of the seed layer YIG as an isolating layer addresses the lattice mismatch issue between the MO material and GaAs. Figure 3d shows the effect of YIG on NRPS. The NRPS sharply decreases with the increase of YIG thickness *H*_YIG_. We opted for a YIG seed layer with a thickness of 50 nm. The thickness allows the growth of superior quality crystalline Ce:YIG films and enables the efficient coupling of the mode’s evanescent field into the Ce:YIG layer. Finally, we chose *H*_Ce:YIG_ = 250 nm, *W*_GaAs_ = 640 nm, and *H*_GaAs_ = 140 nm, resulting in an NRPS of 9000.394 rad/m and a length of 174.53 μm, as shown in Figure 3f. Similar to structure A, the waveguide is in a TM_0_ single-mode condition, as depicted in Figure 3e. This structure is more compact than Structure A, with a smaller footprint compared to most currently demonstrated broadband optical isolators [10,13,22,23,24,25].

### 3.2. Reciprocal Waveguide Structure, MMI Coupler Design

**Figure 4:**
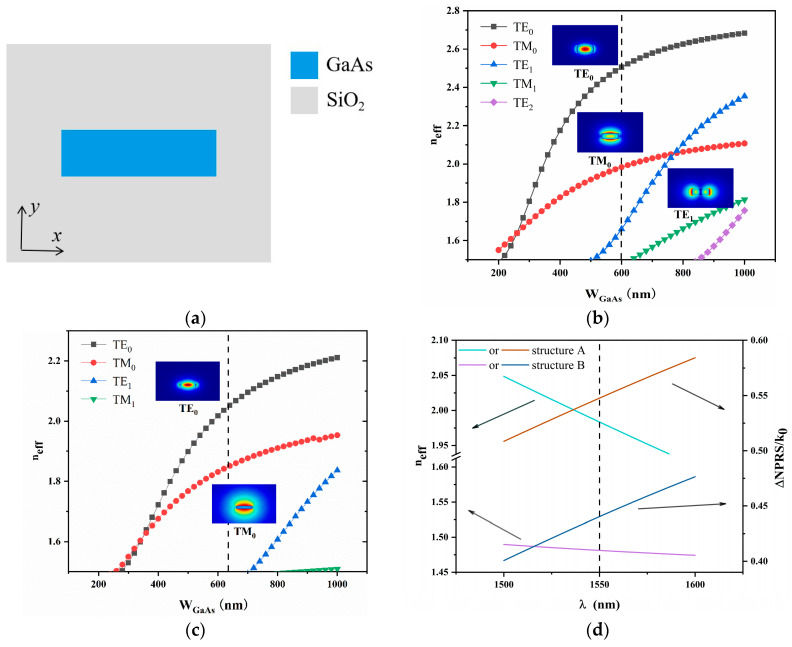
(**a**) Surrounded by SiO_2_ with reciprocal waveguide structure. (**b**) An effective index profile plot of the waveguide modes as a function of the width. At *W*_GaAs_ = 600 nm and *H*_GaAs_ = 260 nm, the mode field distribution with structure A. The dashed line represents that when *W*_GaAs_ = 600 nm, the waveguide is in the TM_0_ mode. (**c**) An effective index profile plot of the waveguide modes as a function of the width. At *W*_GaAs_ = 640 nm and *H*_GaAs_ = 140 nm, the mode field distribution with structure B. The dashed line represents that when *W*_GaAs_ = 640 nm, the waveguide is in the TM_0_ mode. (**d**) The dispersion curves of the two structures are used as a function of wavelength *λ*. The direction of the arrow indicates the direction of linear value extraction.

The reciprocal waveguide shares the same cross-sectional geometry as the NRPS waveguide, illustrated in Figure 4a. The PRS waveguide dimensions for structure A are *W*_GaAs_ = 600 nm and *H*_GaAs_ = 260 nm, while for structure B, they are *W*_GaAs_ = 640 nm and *H*_GaAs_ = 140 nm. Figure 4b,c display the effective index profile plots and mode field distributions of the PRS waveguides for both structures, respectively. The RPS waveguide is the same as the NRPS waveguide and satisfies the TM0 single-mode condition.

The length of the RPS waveguide is calculated as follows:

(4) $$∆L=\frac{\pi /2+2m\pi }{\beta_{0}}$$

where the propagation constants *β*_0_ of structure A and structure B were 8,040,645.743 rad/m and 6,004,449.274 rad/m, respectively. The corresponding ∆L values for different m values are listed in Table 1. When *m* = 0, the difference in arm length between the two structures is 195.36 and 261.61 nm, respectively. Figure 4d displays the effective refractive index *n_eff_* of the reciprocal waveguide or *∆NRPS/k*_0_ vs. wavelength. *n_eff_* decreases with the increase of wavelength, while *∆NRPS/k*_0_ decreases.

**Figure 5:**
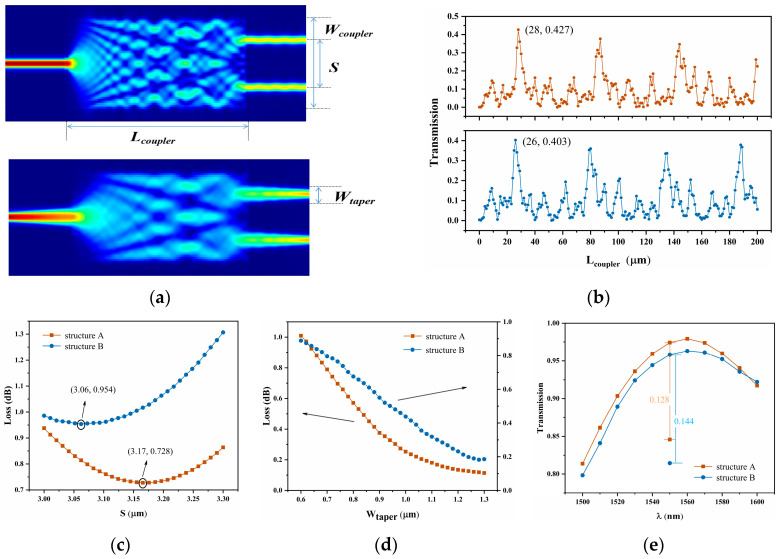
(**a**) MMI is designed with tapered input and output waveguides, and optimal dual self-imaging of structures. (**b**) The function of transmission and coupling region length *L*_coupler_ and its maximum transmittance. (**c**) The effect of different *S* on the loss of two structures. (**d**) Losses using MMI couplers with different cone widths *W*_taper_. (**e**) Spectral pattern with a wavelength of 1500~1600 nm. The single red and blue dots represent the taper-free MMI transmittance at 1500 nm, respectively.

MMI couplers are a fundamental component in high-performance MZIs, showcasing low losses, large optical bandwidth, high thermal stability, and insensitivity to manufacturing tolerances [31]. A 1 × 2 symmetrical 3 dB power divider structure is designed using the eigenmode expansion (EME) method, as shown in Figure 5a. The considered coupler relies on a GaAs waveguide surrounded by SiO_2_. It is worth noting that a wider width of the multimode region leads to higher insertion losses, while being too narrow can result in energy crosstalk between the two output waveguides. The width (*W*_coupler_) of the MMI coupler is set at 6 μm, providing a compact design that prevents undesirable coupling between the two output modes. Furthermore, we meticulously designed the length *L*_coupler_ of the MMI area:

(5) $$L_{coupler}=\frac{3\pi }{\left(\beta_{a}-\beta_{b}\right)}$$

where *β_a_* and *β_b_* are defined as the propagation constants of the base mode TM_0_ and the first-order mode TM_1_, respectively. Figure 5b shows the function relationship between transport and *L*_coupler_. The maximum transmission of the two structures occurs at the length of 28 μm and 26 μm, respectively. Second, the output of the coupler consists of two GaAs waveguides, with a gap of 3.17 μm and 3.06 μm between them, respectively, as shown in Figure 5c. Losses can be minimized by using linear cones on the input/output waveguide, ensuring a good match between the pattern of the input/output waveguide and the interference region. By an EME solver sweep, the *W*_taper_ can be determined. According to the simulation results, the minimum loss is observed at 1.30 μm, as depicted in Figure 5d. The output waveguide narrows gradually using a linear taper from 1.3 µm to 600/640 nm for structures A and B, respectively. The tapering length is set to 10 µm. Figure 5e displays the transmittance in the wavelength range of 1500~1600 nm. With the linear cone, the transmittance for both structures shows an increase of 0.128 and 0.144, respectively, compared to the original at 1550 nm. Consequently, the final losses measure 0.11 and 0.19 dB, respectively.

### 3.3. Insertion Loss, Isolation Bandwidth, Tolerance, and Fabrication Process

Table 2 shows the geometry and material parameters of the MMI isolators of the two proposed structures. Now, we will focus on the typical performance of the device, specifically, insertion loss, isolation bandwidth, and manufacturing tolerances.

The loss of the isolator can be divided into MMI loss, coupling loss between RPS and MO waveguides, RPS waveguide loss, and MO waveguide loss. As previously discussed, the MMI losses for the two structures are 0.11 and 0.19 dB, respectively. The coupling loss is related to modal overlap, and the loss at each connection is approximately 0.25 dB. Hence, the sum loss of the four such coupling structures is about 1 dB. Among them, the propagation loss of the RPS waveguide at a wavelength of 1550 nm is assumed to be *α*_GaAs_ = 4 dB/cm [37], contributing to a device loss of about 0.48 dB. The transmission loss of MO waveguides primarily results from material absorption. The propagation loss of light in the waveguide under TM mode is defined as follows:

(6) $$\alpha_{NRPS}=\alpha_{Ce:YIG/YIG}\times \Gamma_{Ce:YIG/YIG}+\alpha_{GaAs}\times \Gamma_{GaAs}+\alpha_{{SiO}_{2}}\times \Gamma_{{SiO}_{2}}$$

where $\alpha_{Ce:YIG/YIG}$, $\alpha_{GaAs}$, and $\alpha_{{SiO}_{2}}$ represent the losses of the magnetic film, GaAs waveguide, and SiO_2_ waveguide, respectively, while $\Gamma_{Ce:YIG/YIG},\Gamma_{GaAs}\;and\;\Gamma_{{SiO}_{2}}$ denote the confinement factors *Γ* of the composite waveguide, respectively. Among them, according to the literature, *α*_Ce: YIG/YIG_ = 50 dB/cm [13], and *α*_SiO_2__ × *Γ*_SiO_2__ = 0 dB. *Γ* can be calculated as follows:

(7) $$\Gamma =\frac{nc_{0}\varepsilon_{0}\iint \left|E^{2}\right|dxdy}{\iint_{\infty }Re(E\times H^{*})\cdot \tilde{z}\cdot dxdy}$$

The *Γ* of each waveguide are *Γ*_Ce: YIG/YIG_ = 41.15/31.44% and *Γ*_GaAs_ = 62.85/22.70%, respectively. The lengths of the MO waveguides in the two structures are 217.09 and 174.53 μm, resulting in losses of 1.00 and 0.58 dB, respectively. Most losses in structure A come from the core layer, while those in structure B are mainly due to the light absorption of Ce: YIG/YIG. The total insertion losses of the devices are estimated at 2.59 and 2.25 dB, respectively.

**Figure 6:**
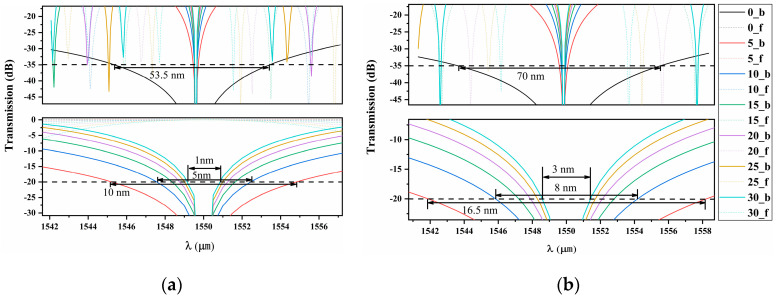
The transmission spectra of the two optical isolators in the forward and backward propagation directions are represented by (**a**,**b**), respectively. Legend 0-b represents the direction of backward propagation when *m* = 0.

In practice, the adjustable length difference between the upper and lower RPS waveguides is employed. For a series of *m* values, the transmission spectrum of 1542 nm~1558 nm will change accordingly, as shown in Figure 6. The solid line represents the forward transmittance, and the dashed line represents the reverse transmittance. Δ*L* significantly impacts the operating bandwidth of the device. A larger length difference renders the device more wavelength-dependent. Additionally, increased Δ*L* results in reduced isolation. When *m* = 0, the maximum isolation bandwidth at 35 dB for both devices is larger than 53.5 and 70 nm, respectively. For structure A, at *m* = 5, the maximum isolation bandwidth at 20 dB is 6.5 nm greater than that of structure B. When *m* = 30, the bandwidth decreases rapidly by 1 and 3 nm, respectively.

We also considered the manufacturing tolerances of the two devices [43]. The sensitivity of the propagation constant to changes in waveguide size has been noted. The manufacturing tolerances significantly impact phase matching, which ultimately affects the isolation bandwidth and loss of the device. Therefore, we simulated the variation of the propagation constant over the different lengths, widths, and thicknesses of the waveguide, and the corresponding results are listed in Table 3. To achieve isolation above 35 dB at a wavelength of 1550 nm, the smallest fabrication tolerance lies in the RPS waveguide length at ±4.41 nm. YIG also has a smaller thickness of ±5 nm. Due to the effect on the coupling coefficient, the core thickness presents a relatively narrow tolerance window of ±8/22 nm. These small manufacturing windows result from the high refractive index of the GaAs waveguides. Conversely, the core width, exhibiting the least sensitivity to NRPS, tolerates variations above ±110 nm for both structures. On the other hand, the MO waveguide length displays a relatively large tolerance window of ±4.89/3.95 μm. To address the challenges posed by stringent device manufacturing tolerances, the introduction of larger Δ*L* or wavelength-tuned waveguides is a potential solution.

Based on the structure of these two devices, we envisage two feasible manufacturing processes. For structure A, the direct bonding technique is proposed. Firstly, a 350 nm Ce:YIG single crystal layer is grown on a (111)-oriented SGGG substrate using the sputter epitaxial growth technique. The surface of the Ce:YIG mold is then activated by exposure to nitrogen plasma. Subsequently, the activated surface at high temperature comes into contact with the upper surface of 260 nm GaAs in a vacuum chamber. An annealing process is carried out under constant and sustained pressure to achieve a strong bond. A similar approach has proven successful for Ce: YIG in SOI [24].

For structure B, it is suggested to integrate the MO material into the semiconductor waveguide using pulsed laser deposition (PLD). A 140 nm GaAs waveguide was fabricated on a SiO_2_ substrate using molecular beam epitaxy (MBE) and reactive ion etching (RIE). Subsequently, using PLD [10,13], 50 nm YIG and 250 nm Ce:YIG thin films were deposited. The substrate remained at room temperature during the deposition process. Thermal vacuum annealing resulted in the formation of Ce:YIG/YIG deposited crystals. In contrast to the PLD technology, the wafer bonding technology allows the preparation of single-crystal garnet in a separate epitaxial growth process. However, the bonded MO material covers a large area, leading to additional device area and absorption loss. PLD offers solutions to these challenges and can be implemented across various device structures.

## 4. Conclusions

In summary, we have proposed two optical isolators based on the GaAs-on-insulator platform. Through the parameter optimization of the MO waveguide, it is demonstrated that the NRPS in the TM mode is insensitive to the *x*-direction gradient distribution of the *H_x_* component. By comparing the waveguides, the NRPS of structure B, containing the YIG seed layer, is 1764.648 rad/m larger than that of structure A, resulting in a shorter length of 174.53 μm. Both structures exhibit an isolation bandwidth exceeding 53.5/70 nm at 35 dB, with losses recorded at 2.59/2.25 dB, respectively. The MMI couplers feature a cone width of 1.3 μm, and the effect of the YIG seed layer on the device performance is discussed. Furthermore, the influence of device geometry on operating bandwidth and manufacturing tolerances is analyzed. Based on the simulation results, a suitable process for these devices is proposed. The robustness exhibited by these devices—such as broad operating bandwidth, high isolation, compact size, low insertion loss, and simplicity in structure—positions them with promising prospects in nonlinear integrated photonic applications. These research findings provide innovative design perspectives for integrated optical isolators based on GaAs-on-insulator.

## Figures and Tables

**Table 1:** The difference in length of the reciprocal waveguide arm under different *m* of the two structures.

*m*	Structure A	Structure B
	Δ*L* (nm)	
0	195.36	261.61
5	4102.49	5493.71
10	8009.63	10,725.81
15	11,916.77	15,957.92
20	15,823.90	21,190.02
25	19,731.04	26,422.12
30	23,638.18	31,654.23

**Table 2:** The relevant structural parameters of the proposed MZI isolators.

Parameter	Structure A	Structure B
	Value	
*H*_GaAs_	260 nm	140 nm
*W*_GaAs_ and Ce:YIG/YIG width	600 nm	640 nm
*H*_Ce:YIG_	350 nm	250 nm
*H*_YIG_	/	50 nm
*L*	217.09 μm	174.53 μm
Δ*L*	195.36 nm	261.61 nm
the *θ_F_* value of Ce: YIG	−5900 deg/cm	
the *θ_F_* value of YIG	/	200 deg/cm
*W*_coupler_	6 μm	
*L*_coupler_	28 μm	26 μm
*S*	3.17 μm	3.06 μm
*W*_taper_	1.3 μm	
Indices of GaAs/Ce:YIG/YIG/SiO_2_/SGGG	3.3/2.22/2.22/1.45/1.97	

**Table 3:** The tolerances of the proposed MZI isolators.

Device Geometries	Structure A		Structure B	
	Value	Tolerances	Value	Tolerances
*H*_GaAs_	260 nm	±8 nm	140 nm	±22 nm
*W*_GaAs_ and Ce:YIG/YIG width	600 nm	±110 nm	640 nm	±157 nm
*H*_Ce:YIG_	350 nm	±72 nm	250 nm	±32 nm
*H*_YIG_	/		50 nm	±5 nm
*L*	217.09 μm	±4.89 μm	174.53 μm	±3.95 μm
Δ*L*	195.36 nm	±4.41 nm	261.61 nm	±5.91 nm

## Data Availability

Data are contained within the article.
